# Pleiotropic effects of regulatory variation in *tan* result in correlation of two pigmentation traits in *Drosophila melanogaster*

**DOI:** 10.1111/mec.14781

**Published:** 2018-07-16

**Authors:** Lukas Endler, Jean‐Michel Gibert, Viola Nolte, Christian Schlötterer

**Affiliations:** ^1^ Institute of Populationsgenetik Vetmeduni Wien Wien Austria; ^2^ CNRS Biologie du Développement Paris Seine‐Institut de Biologie Paris Seine (LBD‐IBPS) Sorbonne Université Paris France

**Keywords:** genetic correlation, genomewide association studies, pigmentation, pleiotropy, pooled sequencing

## Abstract

Traits with a common genetic basis frequently display correlated phenotypic responses to selection or environmental conditions. In *Drosophila melanogaster,* pigmentation of the abdomen and a trident‐shaped region on the thorax are genetically correlated. Here, we used a pooled replicated genomewide association approach (Pool‐GWAS) to identify the genetic basis of variation in thoracic trident pigmentation in two *Drosophila melanogaster* populations. We confirmed the previously reported large effect of *ebony* and the association of the cosmopolitan inversion *In(3R)Payne*. For the first time, we identified *tan* as another major locus contributing to variation in trident pigmentation. Intriguingly, the regulatory variants of *tan* that were most strongly associated with female abdominal pigmentation also showed a strong association with trident pigmentation. We validated this common genetic basis in transgenic assays and found qualitatively similar effects on trident and abdominal pigmentation. Further work is required to determine whether this genetic correlation is favoured by natural selection or reflects a neutral by‐product of a shared regulatory architecture.

## 1 INTRODUCTION

Genetic correlation between quantitative traits is a common phenomenon with many important implications for the evolution of natural populations as well as for breeding of domesticated populations and medical research (Chen & Lübberstedt, 2010; Falconer & Mackay, 1996; Solovieff, Cotsapas, Lee, Purcell, & Smoller, 2013; Wagner & Zhang, 2011). Such correlations can be caused by linkage disequilibrium between distinct causal loci or pleiotropic effects (Falconer & Mackay, 1996; Mackay, Stone, & Ayroles, 2009; Solovieff et al., 2013). The effects of pleiotropy are well‐studied, and theoretical models predict trade‐offs among correlated traits (Barton, 1990; Fisher, 1930; Griswold & Whitlock, 2003; Orr, 2000; Otto, 2004; Roff & Fairbairn, 2007; Wagner et al., 2008). Because classic QTL crosses involve only a moderate number of recombination events, the distinction between linkage and pleiotropy as the cause for correlated traits has traditionally been difficult. Genomewide association studies (GWAS) are a powerful approach to identify pleiotropic genetic variation affecting multiple traits (Solovieff et al., 2013). Nevertheless, the ultimate proof can be only obtained by functional assays demonstrating the pleiotropic effects of candidate variants.

Pigmentation is a classic phenotypic trait and has been extensively studied in *Drosophila*. Because the genetic pathways underlying both the regulation and synthesis of pigments are well described (reviewed in Massey and Wittkopp, 2016), it is possible to link the evolution of this trait with the underlying genetic basis. Across different *Drosophila* species, pigmentation has been associated with various ecologically important traits, such as desiccation and UV resistance, thermal adaptation and cuticle strength, making it an important model for studying adaptation (Bastide, Yassin, Johanning, & Pool, 2014; Rajpurohit, Parkash, & Ramniwas, 2008; True, 2003). Because genes involved in pigment synthesis also influence vision and circadian rhythms or are regulators of spatial patterning (Kronforst et al., 2012; True, 2003), they rely on a modular *cis*‐regulatory architecture to minimize deleterious pleiotropic effects.

In *D. melanogaster,* two pigmentation phenotypes have been intensely studied—pigmentation of the abdominal tergites in female flies and pigmentation of a trident‐shaped region on the dorsal mesothorax—the thoracic trident. While *D. melanogaster* exhibits strong sexual dimorphism in abdominal pigmentation, thoracic pigmentation is similar between sexes (David, Capy, Payant, & Tsakas, 1985). The shape of the trident reflects developmental constraints. Indeed, the trident is complementary to flight muscle attachment sites and the gene *stripe* expressed in flight muscle attachment sites represses multiple pigmentation enzyme coding genes in these sites hence the coexpression of these genes in the trident (Gibert, Mouchel‐Vielh, & Peronnet, 2018). Both abdominal and thoracic trident pigmentations exhibit extensive geographical clines, varying with latitude and altitude (David et al., 1985; Gibert et al., 2004; Parkash, Rajpurohit, & Ramniwas, 2009; Pool & Aquadro, 2007; Telonis‐Scott, Hoffmann, & Sgrò, 2011). This regional variation has been linked to UV irradiation (Bastide et al., 2014) and desiccation resistance (Kalmus, 1941; Parkash et al., 2009; Rajpurohit et al., 2008), indicating that these pigmentation traits could be under selection.

The genetic basis of natural variation in these two pigmentation traits is well‐studied (Bastide et al., 2013; Bickel, Kopp, & Nuzhdin, 2011; Bickel, Schackwitz, Pennacchio, Nuzhdin, & Kopp, 2009; Dembeck et al., 2015; Endler, Betancourt, Nolte, & Schlötterer, 2016; Miyagi, Akiyama, Osada, & Takahashi, 2015; Pool & Aquadro, 2007; Rebeiz, Pool, Kassner, Aquadro, & Carroll, 2009; Robertson, Briscoe, & Louw, 1977; Takahashi, Takahashi, Ueda, & Takano‐Shimizu, 2007; Takahashi & Takano‐Shimizu, 2011), and a handful of key genes involved in either pigment synthesis or spatial patterning have been detected.

Variation of female abdominal pigmentation has been associated with several loci, but the genes *tan, ebony* and *bric‐à‐brac* (*bab*) are probably the strongest contributors (Bastide et al., 2013; Dembeck et al., 2015; Pool & Aquadro, 2007). *tan* and *ebony* encode enzymes involved in melanin synthesis and catalyse reciprocal reactions. A higher activity of *ebony* leads to a lighter colour, while more *tan* activity causes more intense pigmentation (True et al., 2005; Wittkopp, True, & Carroll, 2002). The *bab* locus on the other hand encodes two transcription factors, *bab1* and *bab2*, that are involved in sexual dimorphic spatial patterning in the abdomen (Kopp, Duncan, Godt, & Carroll, 2000; Williams et al., 2008). The most strongly associated variants in genomewide studies (Bastide et al., 2013; Dembeck et al., 2015; Endler et al., 2016) have been found in or close to previously described *cis*‐regulatory regions—the dimorphic anterior element in the first intron of *bab1* (Williams et al., 2008), the enhancer region upstream *ebony* (Rebeiz et al., 2009) and the male‐specific enhancer (*tMSE*) upstream *tan* (Jeong, Rebeiz, Andolfatto, & Werner, 2008). In the two populations used for this study, the three most strongly associated single nucleotide polymorphisms (SNPs) are located in a 208‐bp segment of *tMSE* and their effects on female abdominal pigmentation have been functionally tested with transgenic flies (Gibert, Blanco et al., 2017).

Although trident pigmentation intensity has been studied in several populations from different continents, only variation of *ebony* showed significant association (Miyagi et al., 2015; Takahashi et al., 2007; Telonis‐Scott et al., 2011). Expression levels of *ebony* correlate significantly with intensity of trident pigmentation (Miyagi et al., 2015; Takahashi & Takano‐Shimizu, 2011; Takahashi et al., 2007; Telonis‐Scott et al., 2011; Wittkopp et al., 2002), and an enhancer leading to lighter trident coloration has been found to segregate with the cosmopolitan inversion *In(3R)Payne* (Takahashi & Takano‐Shimizu, 2011). While much of the variation in trident pigmentation could be linked to a 960‐bp core enhancer (abdominal cis‐regulatory element *aCRE*) (Rebeiz et al., 2009; Takahashi & Takano‐Shimizu, 2011), also variants within a 10‐kb window spanning the upstream region and the first intron of *ebony* showed association (Miyagi et al., 2015).

Both female abdominal and trident pigmentations exhibit strong phenotypic plasticity with temperature (Gibert, Moreteau, & David, 2000). Plasticity of abdominal pigmentation has been linked to expression levels of *tan*, with the *tMSE* playing a prominent role. Other enzymes, such as *yellow*, are only contributing to a lesser extent (Gibert, Mouchel‐Vielh, De Castro, & Peronnet, 2016; Gibert, Mouchel‐Vielh, & Peronnet, 2017). In contrast to abdominal pigmentation, the regulation of the trident is less understood. Tentative data for the temperature dependent regulation of *ebony* on the Australian cline suggest that the genetic basis of latitudinal variation in trident pigmentation differs from the one controlling thermal plasticity (Telonis‐Scott et al., 2011).

Artificial selection for female abdominal pigmentation identified a genetic correlation between abdominal and trident pigmentation (Rajpurohit & Gibbs, 2012). The strength of genetic correlation between pigmentation traits varies widely among populations and depends also on the temperature and spacing between the segments scored (Gibert, Moreteau, Scheiner, & David, 1998). To shed more light on the genetic basis of the correlation between trident and female abdominal pigmentation, we performed the first genomewide association study on thoracic trident pigmentation using the same two European populations—from Vienna, Austria, and Bolzano, Italy—that were previously used to map female abdominal pigmentation (Bastide et al., 2013; Endler et al., 2016). Our results do not only corroborate prior studies associating the inversion *In(3R)Payne* and *ebony* with variation of trident pigmentation, but also indicate an important role of *tan* and a few other loci not previously associated with trident pigmentation. The effects of variants around *ebony* are only loosely correlated between female abdominal and trident pigmentation. In contrast, the effects of variants around *tan* on the two traits are highly correlated. We investigated three SNPs within the *tMSE* that are highly associated with female abdominal pigmentation in transgenic flies and observed qualitatively similar effects on both traits, despite the relative contributions being variable.

## 2 MATERIALS AND METHODS

### 2.1 Sample collection, cultivation and pigmentation scoring

Around 30,000 flies were collected in Vienna, Austria (2010), and Bolzano, Italy (2011), as described in Bastide et al. (2013). The flies were split into two replicates for the Viennese population and three for the Italian flies. From each replicate, an *F*_1_ generation was raised in the laboratory, at 18°C for the Viennese population and 25°C for the flies from Bolzano. For each replicate, around 1500 males were phenotyped visually in a dorsal view under a microscope and pigmentation of the thoracic trident was scored on a scale from 0 (very light) to 4 (very dark) similar to David et al. (1985), and for each replicate, one pool with extremely dark and one pool with extremely light individuals was generated for sequencing. In the Vienna population, each pool contained 120 individuals. In the Italian population, the light pool consisted of 100 flies, while only 48–55 individuals with score 3 and 4 (dark and very dark) could be gathered for the dark pools. The Italian pools were further screened for potential *D. simulans* contaminations prior to library preparation, resulting in the removal of one to four individuals for some pools (see Supporting information Table S1).

We used female flies not selected for pigmentation traits to estimate the allele frequencies prior to phenotyping (Endler et al., 2016).

### 2.2 Pooled library preparation and sequencing

Flies from each extreme sample were pooled, and genomic DNA was extracted using a standard high‐salt DNA extraction protocol (Miller, Dykes, & Polesky, 1988) including RNase A treatment.

After fragmentation with a Covaris S2 device (Covaris Inc., Woburn, MA, USA), approximately 1.5 g DNA of each sample were used to prepare paired‐end libraries using the components of the NEBNext^®^ DNA Library Prep Master Mix Set reagents (E6040L) (New England Biolabs, Ipswich, MA, USA) with barcoded TruSeq Single Index adapters (Illumina, San Diego, CA, USA). All libraries were size‐selected for an insert size of 280 bp using AMPureXP beads (Beckman Coulter, Carlsbad, CA, USA) and amplified with 10 PCR cycles. The libraries were sequenced on a HiSeq2000 as 100‐bp paired‐end reads.

### 2.3 Read mapping and filtering

After quality control using fastqc (Andrews, 2010), the sequencing reads were trimmed with PoPoolation and a base quality threshold of 18 and mapped to a reference genome using a Hadoop based computation framework as previously described (Bastide et al., 2013; Kofler, Orozco‐terWengel et al., 2011; Pandey & Schlötterer, 2013). Mapping was performed with BWA aln (v. 0.7.5a‐r405; Li & Durbin, 2009) against the combined genomes of *D. melanogaster* (v. 5.18), *Wolbachia pipientis* (AE017196.1), *Lactobacillus brevis* (CP000416.1), *Acetobacter pasteurianus* (AP011170) and phage *phiX174* (NC_001422.1) without seeding and allowing for two gap openings (‐o 2), an alignment distance threshold leading to loss of <1% of reads assuming a 2% error rate (‐n 0.01), and at most 12 gap extensions (‐e 12 ‐d 12).

After removal of improper pairs, reads mapped with a mapping quality below 20 and duplicated reads using samtools (v. 1.1; Li et al., 2009) and picard Tools (v. 1.104, http://broadinstitute.github.io/picard), the reads were realigned around short insertions and deletions and indels from the *Drosophila* Genetic Reference Panel (Mackay et al., 2012) (DGRP freeze2: ftp://ftp.hgsc.bcm.edu/DGRP/freeze2_Feb_2013/; options RealignerTargetCreator: ‐maxIntervalSize 400 ‐minReadsAtLocus 15; IndelRealigner: ‐entropy 0.05 ‐LOD 3 ‐maxConsensuses 50 ‐greedy 250 ‐model USE_READS) using gatk (v. 3.5.0; McKenna et al., 2010).

The Italian flies were screened for *D. simulans* contamination before library preparation. Nevertheless, we applied the same filtering procedure as described in Bastide et al. (2013) for both populations to remove potential *D. simulans* contamination. In brief, mapped and filtered reads were remapped against a collection of 5 *D. melanogaster* and *D. simulans* genomes using gmap (ver. 2012‐07‐20; Wu & Watanabe, 2005) and all reads mapping to a *D. simulans* genome with a higher mapping quality than to a *D. melanogaster* genome were removed as potential contaminants. The amount of contamination was estimated using 11,000 fixed differences between the two species previously derived from pools of *D. melanogaster* and *D. simulans* (Bastide et al., 2013). The median frequency of the *D. simulans*‐specific markers was below 0.01 in both the Italian and the Austrian pools after decontamination.

popoolation2 (Kofler, Pandey, & Schlötterer, 2011) was used to convert the filtered alignments into a synchronized format and to mask regions 5 base pairs up‐ and downstream of short insertions and deletions as well as repetitive regions (repeatmasker ver. 3.2.8, http://www.repeatmasker.org). Simple low complexity regions were not considered (repeatmasker option: ‐nolow).

After all filtering steps, the coverage varied among replicates between 54‐ and 112‐fold for autosomal chromosomes and correspondingly 50% less for the X chromosome (Supporting nformation Table S2).

For estimating linkage between the three focal SNPs in the *tMSE,* we used read pairs overlapping multiple loci similar to the approach of LDx (Feder, Petrov, & Bergland, 2012). For comparison to other populations, we used data from the second freeze version of the *Drosophila* Genetic Reference Panel (DGRP) (Mackay et al., 2012) and from the *Drosophila* Population Genomics Project (DPGP) phase 2 (multiple African and French lines) and 3 (Zambian lines) downloaded from the *Drosophila* genome nexus (Lack et al., 2015; Pool et al., 2012). The multifasta files from the *Drosophila* genome nexus were converted into the vcf format using the msa2vcf tool of Jvarkit by P. Lindenbaum (https://github.com/lindenb/jvarkit). Only lines genotyped at all three focal SNPs were used.

### 2.4 Association mapping and false detection rate estimation

Association of variants with trident pigmentation was tested using a Cochran–Mantel–Haenszel (CMH) test implemented in popoolation2 as previously described (Bastide et al., 2013; Kofler, Pandey et al., 2011). The CMH test assesses the independence of counts in contingency tables over multiple strata, in our case replicates (Kuritz, Landis, & Koch, 1988). For each position, a 2 × 2 contingency table is constructed based on the counts of reference and alternative alleles in the light and dark pools for each replicate. Odds ratios and confidence intervals were calculated using the mantelhaen.test function of the stats package of R (R Core Team, 2016) and rpy2 (rpy.sourceforge.net).

Only polymorphic sites with a minimum coverage of 15 for each sample, and a minimum overall minor allele count of 8 in each population were considered for testing. The 2% most highly covered sites were excluded from the calculations. All variants were further filtered for a minimum average distance from the ends of supporting reads (average distance >8) and strand bias (max(forward/reverse, reverse/forward) >0.1 or Fisher's exact test on alternative vs. reference strand balance *p‐*value > 10^−2^). Some functions for plotting were adapted from scripts by Stephen Turner (http://www.gettinggeneticsdone.com).

The *p*‐value of the CMH test depends on the counts at each SNP and the sequencing depth of the autosomal chromosomes is on average twice that of the X chromosomes in males. To be able to compare *p‐*values across all chromosomes, we downsampled SNP counts at each autosomal position to 50% for calculation of the *p‐*values (random sampling without replacement), but used full sequencing depths for odds ratios and allele frequency estimates.

To control for multiple testing, we used a false detection rate (FDR) of 0.05 derived from an empirical null distribution as described in Bastide et al. (2013). For annotation of variants, snpeff v4.1 g (Cingolani et al., 2012) was used with ensemble annotations (v.78; Zerbino et al., 2017).

### 2.5 Transgenic *Drosophila* strains for phenotyping

We measured trident pigmentation in transgenic lines harbouring the *tMSE* variants described in (Gibert, Blanco et al., 2017). For constructing these strains, a *tMSE* containing the dark alleles of all three focal SNPs (identified in Bastide et al., 2013) was amplified by PCR from *y* ^*1*^ *w* ^*1118*^ flies and inserted into a pGEMT‐Easy vector (Promega). From this template, all possible combinations of SNPs were derived using site directed plasmid mutagenesis techniques (QuickChange™; Liu & Naismith, 2008). Each *tMSE* with one of the eight combinations of the three SNPs was cloned upstream of an hsp70 minimal promoter and *tan* cDNA. The constructs were inserted at the same genomic location on the third chromosome (86F8) using PhiC31 integrase‐based transgenesis (Bischof, Maeda, Hediger, Karch, & Basler, 2007). These rescue constructs were introduced to an isogenic background mutant for *tan* (allele *t* ^*d07784*^; True et al., 2005). As the transgenes at the homozygous state produced a pigmentation darker than wild type, we produced flies heterozygous for the transgenes and mutant for *tan* by crossing flies homozygous for the transgenes and for the *t* ^*d07784*^ allele with the *t* ^*d07784*^ stock. The transgenic flies used for the phenotyping were grown at 25°C.

### 2.6 Quantification of thoracic trident pigmentation

Flies were immersed in 75% Ethanol and pinned through the abdomen on a silicon substrate. Their thoraces were imaged with a binocular equipped with Leica DC480 digital camera using the Leica IM50 Image Manager software. An annular lamp was used to ensure homogeneous lighting. All pictures were taken with identical settings during the same session. To quantify the intensity of the trident, we delimited the trident using a polygon based on the suture anterior to the scutellum and the base of each dorsocentral macrochaetes using imagej (Rasband, 2016) (see Supporting information Figure S7). We measured the mean intensity of the pixels included in this polygon with the function “measure” of imagej. The obtained values were subtracted from 255 to get values comprised between 0 (white) and 255 (black). The trident of ten males was quantified for each *tMSE* haplotype.

### 2.7 In silico Pool‐GWAS simulations

The effect of the haplotype structure of the three variants in the *tMSE* on Pool‐GWAS experiments was simulated using a custom script in R v. 3.3.2 (R Core Team, 2016). For each in silico experiment, five replicates with 1500 individual each were created with haplotypes reflecting the frequencies estimated in the pooled European base populations using paired‐end read data (Supporting information Table S4). Each of the five occurring haplotypes was assigned the median of the phenotypic values measured for the corresponding transgenic individuals as a base pigmentation score. Assuming a heritability of *h* ^2^ = 0.2, a normally distributed noise term was added to the base pigmentation score and the individuals were ranked according to their pigmentation. The allele counts of the lightest 100 and the darkest 75 of each replicate were contrasted using the mantelhaen.test function of R.

### 2.8 Statistics

All statistical calculations were performed using R v. 3.3.2 (R Core Team, 2016).

For fitting the full factorial three‐way ANOVA, the Anova function from the library car was used with Type III sums of squares (SS) and zero sum constraints on factor contrasts (option contr.sum) as previously described (Gibert, Blanco et al., 2017). Effect sizes were estimated as *η* ^2^ = SS_Effect_/SS_Total_.

To identify significant associations between cosmopolitan inversions and trident pigmentation, the frequency of each inversion in each pool was estimated as the median frequency of marker SNPs (Kapun, van Schalkwyk, McAllister, Flatt, & Schlötterer, 2013). A linear model was fitted to the arcsine square‐root transformed frequency (arcsine(frequency^0.5^)) considering the population (Vienna and Bolzano) and the extreme pool (light and dark) as categorical predictors. The model was fitted using the lm function of R and the *p*‐value for the coefficient of the extreme pool corrected for multiple testing using the Bonferroni method of the p.adjust function.

## 3 RESULTS

We used the same two European populations from Vienna, Austria, and Bolzano, Italy, that were previously used to characterize female abdominal pigmentation (Bastide et al., 2013). Differentiation between the populations is low (*F*_ST_ = 0.0107, 95% C.I.: [0.0105, 0.0110]; see Endler et al., 2016) and we combined the samples from the two populations as in the previous study unless stated otherwise. After mapping and filtering, around 2.6 million SNPs were identified in the extreme pools.

### 3.1 Inversions associated with trident pigmentation

**Figure 1:**
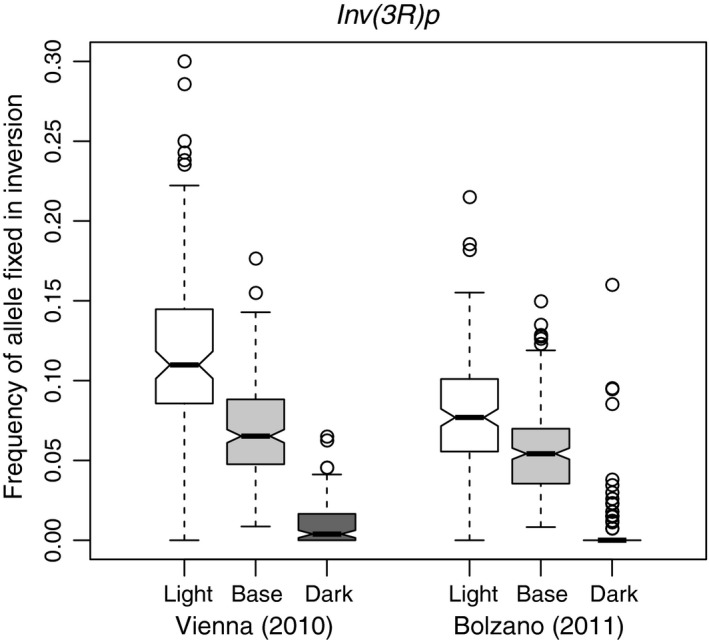
Frequency of the cosmopolitan inversion *In(3R)Payne* in pooled individuals with extreme pigmentation (white: very light, dark grey: very dark) and the base population (light grey) of the populations from Vienna and Bolzano. The inversion frequencies were estimated using 62 marker SNPs for the inversion (Kapun et al., 2013). All replicates were combined for easier interpretation

Multiple studies of natural populations associated variation in trident pigmentation with the *ebony* gene (Miyagi et al., 2015; Takahashi et al., 2007; Telonis‐Scott et al., 2011). Some enhancer variants of ebony causing lighter pigmentation are in full linkage with the cosmopolitan inversion *In(3R)Payne* (Takahashi & Takano‐Shimizu, 2011). Therefore, we estimated the frequency of cosmopolitan inversions using inversion‐specific SNPs (Kapun et al., 2013). For each inversion, the median frequency of multiple markers was taken as an estimate of the frequency in the pool. For association testing, we used a linear model with the populations (Vienna and Bolzano) and pigmentation (light and dark) as categorical predictors and fitted them to the arcsine transformed inversion frequencies. Consistent with (Takahashi & Takano‐Shimizu, 2011) *In(3R)Payne* was significantly associated with trident pigmentation (*p‐*value < 0.001), with higher frequencies in the light pools and being almost absent in the dark pools (light: 9.2 ± 3.9%, dark:0.2 ± 0.3%, see Figure 1). Among the other inversions, we detected a significant association only for *In(3R)c*, which partially overlaps *In(3R)Payne* (*p‐*value = 0.027). However, the frequency differences between light and dark pools were not as marked as *In(3R)Payne* (Supporting information Figure S1).

### 3.2 SNPs associated with trident pigmentation

**Figure 2:**
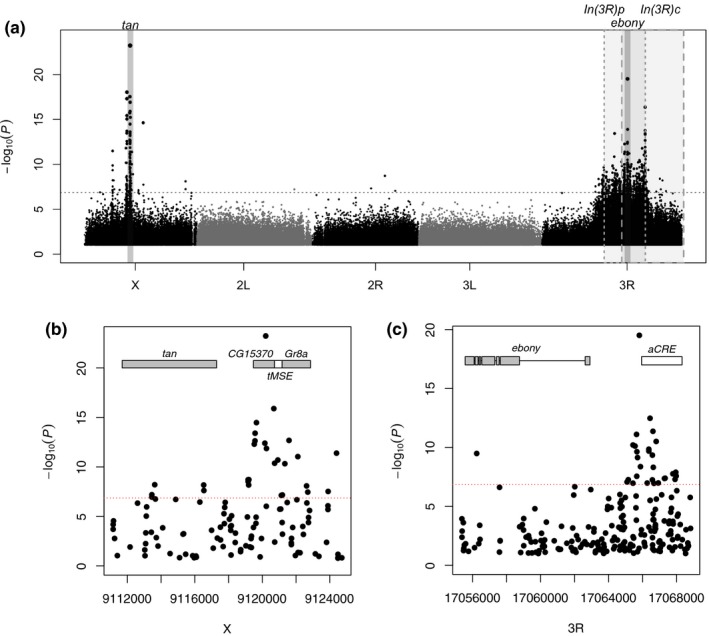
Pool_GWAS for trident pigmentation: (a) Manhattan plot showing the association of SNPs with trident pigmentation in the combined populations from Vienna and Bolzano. The *y*‐axis shows the negative decadic logarithm of the *p*‐value of the CMH test, with the horizontal dotted line indicating the empirical 5% FDR threshold. The region around *tan* and *ebony* are highlighted in dark grey. The grey rectangles indicate the location of the two significantly associated cosmopolitan inversions. To make *p‐*values comparable across the genome, SNP counts at autosomal loci were downsampled to 50%. The lower panel shows close‐ups around the *tan* (b) and *ebony* (c) genes with grey boxes indicating exons, connecting lines introns. The male‐specific enhancer region (*tMSE*) and the abdominal cis‐regulatory element (*aCRE*) are depicted as white rectangles [Colour figure can be viewed at http://wileyonlinelibrary.com]

We studied the association of SNP variants with trident pigmentation by contrasting their allele frequencies in the light and dark pools using a Cochran–Mantel–Haenszel (CMH) test. With a 5% false discovery rate (FDR), we find a large number of SNPs (739) to be significantly associated with trident pigmentation. Most of them (578) are located on chromosome 3R within or close to the region of the cosmopolitan inversion *In(3R)Payne* (see Figure 2a and Supporting information Table S3).

**Figure 3:**
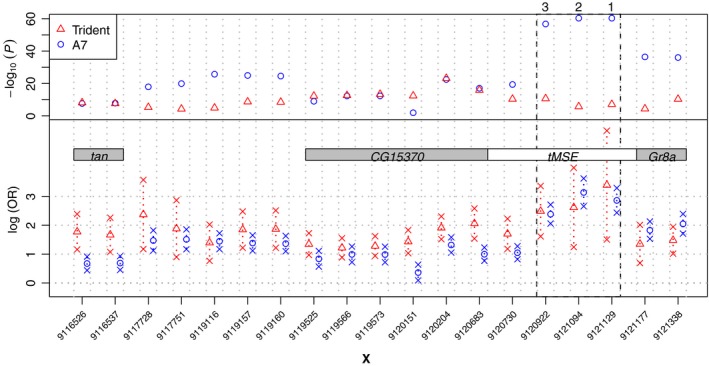
Comparison of the most highly associated SNPs in the trident Pool‐GWAS (triangles) with the results of the abdominal pigmentation GWAS (A7, circles; Endler et al., 2016) around the *tan* locus. In the upper panel the negative decadic logarithm of the *p‐values* of the SNPs are shown, in the lower the natural logarithm of the estimated odds ratio of the dark‐allele counts in the dark versus the light pools is shown (log(OR)) as a proxy for effect size. Grey rectangles indicate the location of genes, and the male‐specific enhancer (*tMSE*) is shown in white. Only SNPs with a *p*‐value below 10^−6.8^ for trident or 10^−14^ for abdominal pigmentation are displayed. The dashed lines indicate the position the three SNPs (SNP1: X:9121129, SNP2: X:9121094 and SNP3: X:9120922) used for the transgenic assays [Colour figure can be viewed at http://wileyonlinelibrary.com]

The SNPs with the strongest association map upstream of the *tan* gene (Figure 2 and 3, Supporting information Figure S3). While *tan* was previously associated with variation in pigmentation of abdominal tergites (Bastide et al., 2013; Dembeck et al., 2015; Endler et al., 2016), this is the first time that it can also be associated with variation in trident pigmentation. The most significant variant is a missense mutation in the gene *CG15370*, which is located directly upstream of *tan*. The same SNP was also strongly associated with female abdominal pigmentation (Bastide et al., 2013; Endler et al., 2016). Furthermore, a group of three closely linked SNPs within the male‐specific enhancer element (*tMSE*), which were most strongly associated with female abdominal pigmentation in multiple populations (Bastide et al., 2013; Dembeck et al., 2015; Endler et al., 2016), show strong effects for trident pigmentation too. Only two of them (X:9120922 and 9121129; Dmel. rel. 5) reach significance (Supporting information Figure S3).

Another group of strongly associated variants is located around the ebony locus. It partially overlaps with previously described enhancer elements for abdominal pigmentation (abdominal Cis‐Regulatory‐Element, *aCRE*) (Pool & Aquadro, 2007; Rebeiz et al., 2009) and trident pigmentation (Takahashi & Takano‐Shimizu, 2011; Takahashi et al., 2007) (Figure 2c and Supporting information Figure S4). However, due to the strong signal produced by the inversion *In(3R)Payne*, these results are hard to interpret. The whole genomic region covered by the inversion shows an elevated background and multiple peaks of significant variants, especially close to the inversion breakpoints. As recombination rates are lowest around the breakpoints, variants located there are more likely to be in linkage disequilibrium (Stevison, Hoehn, & Noor, 2011). Indeed, a number SNPs with low *p*‐values close to the breakpoints of *In(3R)Payne* have also been identified as inversion‐specific markers (Kapun et al., 2013). Further evidence for the strong influence of *In(3R)Payne* comes from the comparison of the inversion and the strongly associated SNPs. Both differ about 10% in frequency between light and dark pools, which suggests linkage disequilibrium, but not necessary causality. In contrast, some candidate SNPs around the ebony locus differ up to 50% in frequency (Supporting information Figure S8). With no corresponding effect in this genomic region for abdominal pigmentation (Bastide et al., 2013), we conclude that trident‐specific enhancer haplotypes are associated with the inversion, but that the association is not complete, such that a strong signal around *ebony* could still be distinguished from the inversion signal.

In addition to the region covered by *In(3R)Payne* and *tan*, we find several significant associations on the X chromosome that were not previously linked to trident or abdominal pigmentation (see Supporting information Figure S6 and Supporting information Table S3). The second most significant region on the X chromosome shows an effect only in the Italian population (Supporting information Figure S4 and Supporting information Table S3). The significant variants segregate at very low frequencies in both populations – <2% in the Viennese flies and at around 5% in the ones from Bolzano (Supporting information Table S3). Because the power of the Pool‐GWAS decreases with allele frequency and the number of replicates (Bastide et al., 2013; Endler et al., 2016) and the logOR suggest similar effects in both populations, it is possible that the difference between populations reflects power rather than an effect of different assaying temperatures or no biological effect in the Viennese flies. The most significant variants map to the coding region of the gene *Crag*, a guanine exchange factor involved in epithelial structure formation and protein trafficking (Denef, Chen, Weeks, Barcelo, & Schüpbach, 2008; Xiong et al., 2012). Knockdown of *Crag* results in body colour defects in the dorsal mesothorax (Mummery‐Widmer et al., 2009) corroborating a potential role in trident pigmentation.

Another region with highly associated SNPs is located around position 9,052,000 of the X chromosome with synonymous variants in the genes *Bap111/daloa* and *Ost48*. Neither of these genes has been linked to pigmentation before. *Bap111* is a subunit in the *Brahma* Swi/Snf like chromatin remodelling complex (Papoulas et al., 2001), *Ost48* is involved in protein glycosylation and cellular secretory pathways (Kondylis, Tang, Fuchs, Boutros, & Rabouille, 2011). While both genes could have an indirect role in regulation of the transcription or excretion of enzymes involved in pigment formation, there is no prior evidence in this direction. Similar to the variants at *Crag*, the variants only show significant association in the Italian samples with consistent effects and very low allele frequencies in the Viennese population. A less significant peak is located around position 5,600,000 encompassing the genes *CG15771* and *CG15772*. While both genes are not well‐characterized, knockdown of *CG15772* changes pigmentation in the dorsal mesothorax (Mummery‐Widmer et al., 2009). Another gene on the X chromosome harbouring a significantly associated missense variant is Megalin (*Mgl*), a gene involved in *Yellow* clearance and in cuticle melanization (Riedel, Vorkel, & Eaton, 2011). Further significant SNPs lie on chromosome 2L and 2R although not close to any obvious candidates involved in regulation of pigmentation (Supporting information Table S3).

### 3.3 Comparison with female abdominal pigmentation

As the same populations were used to map the genetic basis of female abdominal pigmentation (Bastide et al., 2013; Endler et al., 2016) and trident pigmentation, we can readily compare variants associated with both traits around the *ebony* and *tan* region. We use logOR of alleles between the light and dark pool as a proxy for their effect as it has been shown to be highly correlated with allelic effects in simulations (Bastide et al., 2013; Endler et al., 2016). The variants around *ebony* that were most strongly associated with female abdominal pigmentation show consistent effects for the trident—that is the same light and dark alleles and similar relative logOR. However, this is not the case for variants strongly associated with trident pigmentation. The most significant SNPs for trident pigmentation, which also show the largest effects, show little association with female abdominal pigmentation and smaller or inconsistent effects. This also applies to variants in regions that influence abdominal pigmentation in other populations, especially the *aCRE* of *ebony* (Miyagi et al., 2015; Pool & Aquadro, 2007; Rebeiz et al., 2009; Takahashi & Takano‐Shimizu, 2011). Overall, the logOR of the 38 SNPs around *ebony*, which are significant for either trident or abdominal pigmentation, are negatively correlated (Pearson's correlation: −0.71), suggesting that variants around *ebony* do not contribute strongly to the correlation of the two traits in our populations.

Comparing the highly associated SNPs upstream of *tan* between the two traits, we find a substantial overlap and a strong correlation of effects (17 SNPs significantly associated with either trait; Pearson's correlation: 0.72, *p‐*value = 0.0012, *df* = 15, *t* = 3.99) (Figure 3). In both studies, the three closely linked SNPs within the *tMSE* show the largest effect in this region, suggesting that these variants may regulate both traits.

### 3.4 Effects of variants around *tan*

We used transgenic flies (Gibert, Blanco et al., 2017) to investigate the effect of the three *tMSE* SNPs on trident pigmentation. These transgenic flies contain the *tMSE* enhancer with one of the eight combinations of the alleles of the three SNPs fused to the *tan* cDNA under an *hsp70* minimal promoter. All measurements of pigmentation were performed in males with a loss‐of‐function mutant *tan* allele (*t* ^*d07784*^; True et al., 2005) as described in (Gibert, Blanco et al., 2017). To be consistent we follow the previous naming convention (Bastide et al., 2013; Gibert, Blanco et al., 2017): SNP1: X‐9121129, SNP2: X‐9121094, SNP3:X‐9120922, which also distinguishes alleles dark (D) and light (L) according to the effect predicted by the association studies.

**Figure 4:**
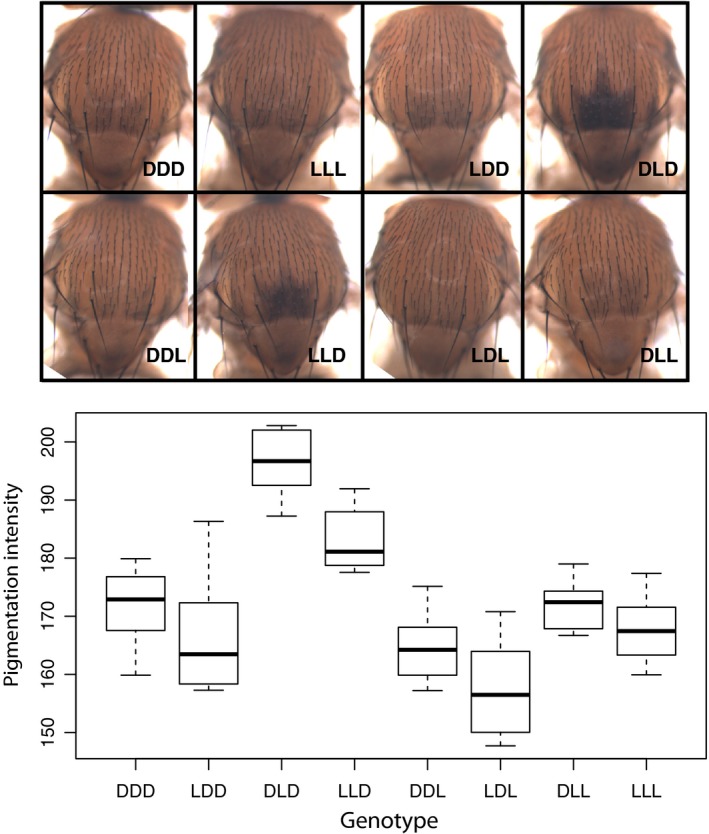
Influence of three SNPs in *tMSE* evaluated in a transgenic assay. Thoracic trident pigmentation in transgenic flies with all different combinations of three regulatory SNPs (upper panel). Boxplots of the measured trident pigmentation for the different genotypes (10 individuals per genotype). D and L indicate the dark and light allele as predicted in (Bastide et al., 2013). The position of the letters indicates SNP1, SNP2 or SNP3 as defined in the text. Pigmentation intensity is calculated as 255 minus the mean pixel intensity as described in 2 [Colour figure can be viewed at http://wileyonlinelibrary.com]

We find pronounced differences in pigmentation intensity between the different combinations of alleles (Figure 4, Table 1). As noted for abdominal pigmentation in females (Gibert, Blanco et al., 2017), the effect of SNP2 is opposite to the effect predicted by the association study. For this SNP, the predicted dark allele decreases pigmentation strength, and the darkest phenotype is produced by a combination of the light allele of SNP2 with the dark alleles of the other SNPs (DLD in Figure 4). Similar to female abdominal pigmentation, the main effects of the three SNPs explain 70% of the observed variation. While the epistatic interaction between SNP2 and SNP3 is statistically significant, it only explains 5% of the total variation. Compared to female abdominal pigmentation, the ranking of the SNPs is markedly different for trident pigmentation in males. SNP2 and SNP3 explain 4 and 3.5 times as much of the observed variation in trident pigmentation as SNP1, which shows by far the strongest effect on female abdominal pigmentation (Table 1). Nevertheless, in the trident GWAS SNP1 and SNP3 exhibit significant association (*p*‐values 2 × 10^−11^ and 7.3 × 10^−8^, respectively). SNP2, on the other hand, which shows the strongest effect in the transgenic flies, does not reach significance (*p*‐value = 1.8 × 10^−6^). This surprising discrepancy could at least partially be explained by the haplotypic configurations inferred from the reads in the combined base population (Supporting information Tables S4 and S5). SNP1 and SNP2 only occur in the LL and DD combination, which has a smaller combined effect than SNP2 alone. Notably, the strong linkage disequilibrium between the two SNPs is not restricted to the two European populations from this study, but can be also found in the DGRP and DPGP2/3 data sets (Tables S4 and S5) and was observed in flies from South Africa (Endler et al., 2016).

**Table 1:** Comparison of the results of the three‐way ANOVA on male trident pigmentation with the results for abdominal cuticle pigmentation in female flies (taken from: Gibert, Blanco et al. (2017))

	A5		A6		A7		Trident	
	*p‐*value	*η* ^2^	*p‐*value	*η* ^2^	*p‐*value	*η* ^2^	*p‐*value	*η* ^2^
1	**<0.001**	**0.422**	**<0.001**	**0.533**	**<0.001**	**0.295**	**<0.001**	**0.082**
2	**<0.001**	**0.059**	**<0.001**	**0.097**	**<0.001**	**0.132**	**<0.001**	**0.328**
3	**<0.001**	**0.130**	**<0.001**	**0.081**	**<0.001**	**0.180**	**<0.001**	**0.289**
1 × 2	0.206	0.004	**<0.001**	**0.025**	0.394	0.002	0.400	0.002
1 × 3	0.260	0.003	**<0.001**	**0.033**	0.074	0.009	0.224	0.005
2 × 3	**<0.001**	**0.058**	**0.013**	**0.010**	**<0.001**	**0.047**	**<0.001**	**0.052**
1 × 2 × 3	**<0.001**	**0.045**	**<0.001**	**0.042**	0.025	0.015	0.082	0.010
Residuals		0.280		0.179		0.320		0.232

Notes

Significant effects (*p‐*value < 0.05) are in bold. A5, A6 & A7: abdominal segments 5, 6 & 7 in females, 1, 2 & 3: three most significant SNPs in the abdominal pigmentation Pool‐GWAS (Bastide et al., 2013). *η* ^2^ is a measure of effect size derived from the ANOVA as the proportion of total variance explained by the specific factor or interaction.

We substantiated the influence of linkage disequilibrium on the estimated effect of SNP2 in the association study and the transgenic assays by computer simulations. We used the haplotype frequencies in the European populations obtained from paired‐end reads (Supporting information Table S4) and the pigmentation scores from the transgenic assays (Supporting information Table S6) as input parameters for the simulations. Because the three SNPs probably account only for a fraction of the phenotypic variation, we used a low heritability of *h* ^2^ = 0.2. We simulated five replicates of 1500 individuals and the SNPs in a light pool of 100 individuals and a dark pool of 75 individuals was subjected to CMH tests. Consistent with the proposed influence of the haplotype structure, we inferred for SNP2 the same direction of the effect as in the experimental Pool‐Seq data, which is opposite to the transgenic assays (Supporting information Figure S9). Unlike the experimental Pool‐GWAS results, in simulations, SNP3 had by far the largest effect and a low median *p*‐value. We attribute this discrepancy to the fact that the transgenic constructs only contain the *tMSE* region and no other regions upstream *tan* that could be involved in its regulation.

## 4 DISCUSSION

In the first genomewide association study of trident pigmentation in *D. melanogaster*, we validated the previously described association between the inversion *In(3R)Payne* and the *ebony* locus (Pool & Aquadro, 2007; Takahashi & Takano‐Shimizu, 2011; Takahashi et al., 2007; Wittkopp et al., 2002). While the inversion is associated with a light trident (Takahashi & Takano‐Shimizu, 2011), it changes <10% in frequency between the light and dark extreme pools. Thus, the most strongly associated variants around *ebony* segregate independently of the inversion. Nevertheless, the genomic region covered by the inversion—especially at the breakpoints—results in an elevated background of associated SNPs, which makes interpretation of results in this region difficult and distorts the false positive rate estimation. We would like to point out that such inversions are a general problem for mapping causative alleles as the number of recombination events is reduced. While Pool‐GWAS is not well suited for mapping causative variants when recombination is suppressed, it is possible to map causative alleles by the sequencing of recombinant genotypes provided that they are available in sufficient quantity.

The genomic region around *ebony* and *In(3R)Payne* shows marked differences for the trident and abdominal pigmentation GWAS. In contrast to its role in trident pigmentation, the inversion is not associated with female abdominal pigmentation. While variants around *ebony*, which are associated with abdominal pigmentation, also influence trident pigmentation, the variants most strongly associated with trident pigmentation have almost no effect on the female abdominal pigmentation GWAS (Supporting information Figure S5). We conclude that the regulatory variants segregating with *In(3R)payne* have a stronger effect on trident than on female abdominal pigmentation and that some variants around *ebony* are exclusively affecting trident pigmentation.

Apart from the variants within *In(3R)Payne,* we mainly find associations on the X chromosome. The high density of candidate regions on the X chromosome is remarkable. Since we only scored males, it is possible that hemizygosity of the X chromosome provides penetrance to low frequency recessive variants, which would be difficult to detect in females. A few of these regions overlap genes that have been connected to pigmentation phenotypes—though not in association studies—such as *Crag*,* CG15772* and *Megalin*, illustrating the power of the Pool‐GWAS approach.

In our study, we combined data from two populations, which were maintained at two different temperatures. Because the CMH test is designed for consistent signals across replicates, this is a conservative approach targeted to find strong effects that are not dependent on the population surveyed or the culture conditions. The drawback of this approach is that population‐specific effects are difficult to disentangle from temperature‐specific effects. For some of the variants on the X chromosomes outside *tan*, most notably around *Crag*,* Bap11* and *mgl*, we note that the significant effects were detected in the Italian population, which has more replicates and a higher frequency of the candidate alleles. In combination with similar logOR in both populations, this suggests that the different significances could reflect power, rather than biological differences between the populations. Nevertheless, further follow‐up experiments are needed to resolve this question.

The most surprising result of our study is that *tan*, which was previously only known to modulate variation in female abdominal pigmentation, also harbours segregating variation affecting trident pigmentation. The effect of *tan* is more strongly correlated between abdominal and trident pigmentation than for *ebony*. This indicates a common regulatory architecture of the two traits at *tan* but not *ebony*. Further inspection of the three SNPs in the *tMSE*, which are most highly associated with abdominal pigmentation in females, showed that all three SNPs also influence trident pigmentation in males. While these effects are qualitatively consistent with the ones on abdominal pigmentation, they differ drastically in the ranking of their effect size. Contrasting the effect size from Pool‐GWAS to results from transgenic constructs showed that the SNP with the strongest effect in transgenes exhibits the lowest association with the trait. Similar to female abdominal pigmentation, where the effect of SNP2 was estimated in the wrong direction, we also attribute this discrepancy to the linkage structure in the mapping population. This is further supported by in silico simulations, which show that the pronounced haplotype structure of the three regulatory SNPs leads to the apparent reversal of the allelic effect of SNP2 and the strongest association for SNP3. We conclude that allelic effects estimated from GWAS studies can be incorrect when multiple sites are contributing to the phenotype and their linkage structure is not considered.

The genes *tan* and *ebony* encode enzymes with antagonist activities. Thus, the amount of melanin produced is directly linked to the expression ratio of *ebony* and *tan*, and as *ebony* is epistatic over *tan* (because Ebony's product is Tan's substrate), *ebony* must be expressed for *tan* to have an effect (Gibert et al., 2016). In situ hybridizations have shown that both genes are expressed in female abdominal epidermis (Gibert et al., 2016; Rebeiz et al., 2009). The expression of *ebony* in abdominal epidermis was shown to be regulated by the combination of activator and silencer elements distantly located (Rebeiz et al., 2009). The *ebony* regulatory sequences regulating its expression in the trident overlap with the activator element (Rebeiz et al., 2009). For *tan*, the *tMSE* element drives GFP expression in abdominal epidermis in males (Jeong et al., 2008) and females (Gibert et al., 2016) as well as in the trident (Gibert et al., 2018). This explains how SNPs located in this enhancer can affect both female abdominal pigmentation and the trident.

It is well established that the modularity of enhancers allow different body parts to be controlled independently by the same genes providing an important factor in the evolution of morphology (reviewed in Prud'homme, Gompel, and Carroll (2007)). Correlation of traits can lead to restrictions and trade‐offs in adaptation (Chen & Lübberstedt, 2010; Falconer & Mackay, 1996; Wagner & Zhang, 2011). Consistent with previous studies (Rajpurohit & Gibbs, 2012), we show that pigmentation of the thoracic trident and the female abdomen are not independent, but genetically correlated. Demonstrating that the two pigmentation phenotypes are influenced by the same regulatory sequence in *tMSE* with the same causative SNPs segregating in natural populations, our study adds to the understanding of the genetic basis of correlated traits.

## AUTHOR CONTRIBUTIONS

C.S. and V.N. conceived the study. L.E. and J.M.G. analysed the data. J.M.G. and V.N. performed the experiments. All authors contributed to writing the manuscript.

## DATA ACCESSIBILITY

Custom scripts are available under https://github.com/luenling/TridentPigmentation2018. Sequencing reads are available at the European Nucleotide Archive (ENA) under the accessions PRJEB3292 (reference populations) and PRJEB25181 (trident pigmentation extreme pools).
