# Supplementary material for: Pleiotropic effects of regulatory variation in tan result in correlation of two pigmentation traits in Drosophila melanogaster
Source: Mol Ecol. 2018 Jul 16;27(16):3207–18. doi: 10.1111/mec.14781 (PMC6120501; doi:10.1111/mec.14781)
Supplement: Supplementary file 1 [file MEC-27-3207-s001.pdf]

MOLECULAR ECOLOGY

Supplemental Information for:

Pleiotropic effects of regulatory variation in *tan* result in correlation of two pigmentation traits in *Drosophila melanogaster*

Lukas Endler, Jean-Michel Gibert, Viola Nolte, and Christian Schlötterer

Table of Contents:

**Table S1 & S2**	Page 2
**Table S4 & S5**	Page 3
**Figure S1**	Page 4
**Figure S2**	Page 5
**Figure S3**	Page 6
**Figure S4**	Page 7
**Figure S5**	Page 8
**Figure S6**	Page 9
**Figure S7**	Page 10
**Figure S8**	Page 11
**Figure S9**	Page 12

MOLECULAR ECOLOGY

Vienna					
Replicate 1		Replicate 2			
L	D	L	D		
119	120	120	120		
Bolzano					
Replicate 1		Replicate 3		Replicate 4	
L	D	L	D	L	D
99	54	96	54	100	48

Table S1: Number of individuals with extreme pigmentation (L: very light, D: very dark) pooled for Pool-GWAS analysis.

	Vienna				Bolzano					
	Replicate 1		Replicate 2		Replicate 1		Replicate 2		Replicate 3	
	L	D	L	D	L	D	L	D	L	D
Autosomes	54	88	99	80	107	97	72	99	112	83
X chrom	27	43	49	40	54	48	36	50	56	41

Table S2: Sequencing depths for autosomal and X chromosomes for different replicates used in the study. L: very light trident, D; very dark trident

MOLECULAR ECOLOGY

	Eu		DGRP		DPGP2		DPGP3	
**Haplotype**	**Count**	**Freq.**	**Count**	**Freq.**	**Count**	**Freq.**	**Count**	**Freq.**
**D.D.D**	210	75.00%	88	53.66%	13	10.08%	3	1.57%
**D.D.L**	25	8.93%	27	16.46%	64	49.61%	52	27.23%
**L.L.D**	6	2.14%	1	0.61%	1	0.78%	0	0.00%
**L.L.L**	38	13.57%	39	23.78%	46	35.66%	132	69.11%
**L.D.D**	1	0.36%	8	4.88%	0	0.00%	0	0.00%
**L.D.L**	0	0.00%	0	0.00%	4	3.10%	2	1.05%
**D.L.D**	0	0.00%	0	0.00%	0	0.00%	0	0.00%
**D.L.L**	0	0.00%	1	0.61%	1	0.78%	2	1.05%
**L.*.***		18.00%		29.20%		39.53%		70.16%
.L.		17.00%		25.00%		37.21%		70.16%
***.*.L**		22.00%		40.85%		89.15%		98.43%

Table S4: Frequencies of haplotypes of the 3 SNPs in the *tMSE* and their light alleles (last three rows) in the European populations used for this study (**Eu**), and in lines from **DGRP** (164 lines) and **DPGP2** (129 African and French lines) and **DPGP3** (132 lines from Zambia). The haplotype are sequences of the dark (D) or light (L) allele for SNP1, 2 and 3 as indicated in the main text. The **Count** column gives the number of read pairs supporting each haplotype in the combined European base, or the number of isofemale lines with the indicated haplotypes. Allele frequencies for the European base population were estimated as averages of all replicates, and not only from the read pairs spanning all three loci.

	Eu	DGRP	DPGP2	DPGP3
**r^2(1,2)**	0.97	0.75	0.84	0.9
**r^2(1,3)**	0.44	0.27	0.01	0.04
**r^2(2,3)**	0.47	0.44	0.01	0.04

Table S5: Pairwise linkage disequilibrium estimates (**r^2**) for all pairs of the three SNPs (1,2, 1,3 and 2,3) in the *tMSE* in the populations mentioned in supplementary Table S4.

MOLECULAR ECOLOGY

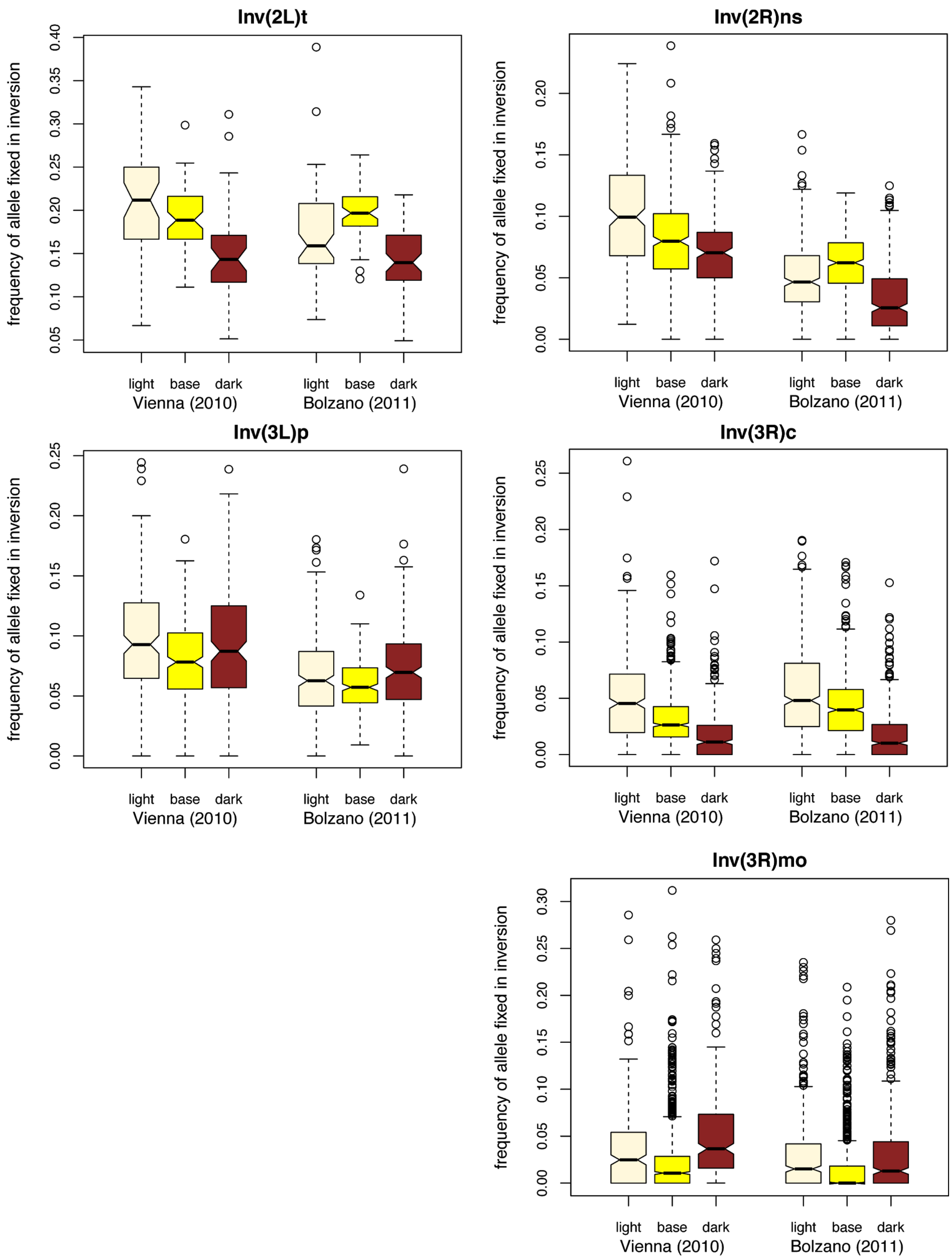

Figure S1: Frequencies of alleles fixed in different cosmopolitan inversions as in Figure 1. The colors indicate the pools with extreme pigmentation (ivory: very light, brown: very dark)

MOLECULAR ECOLOGY

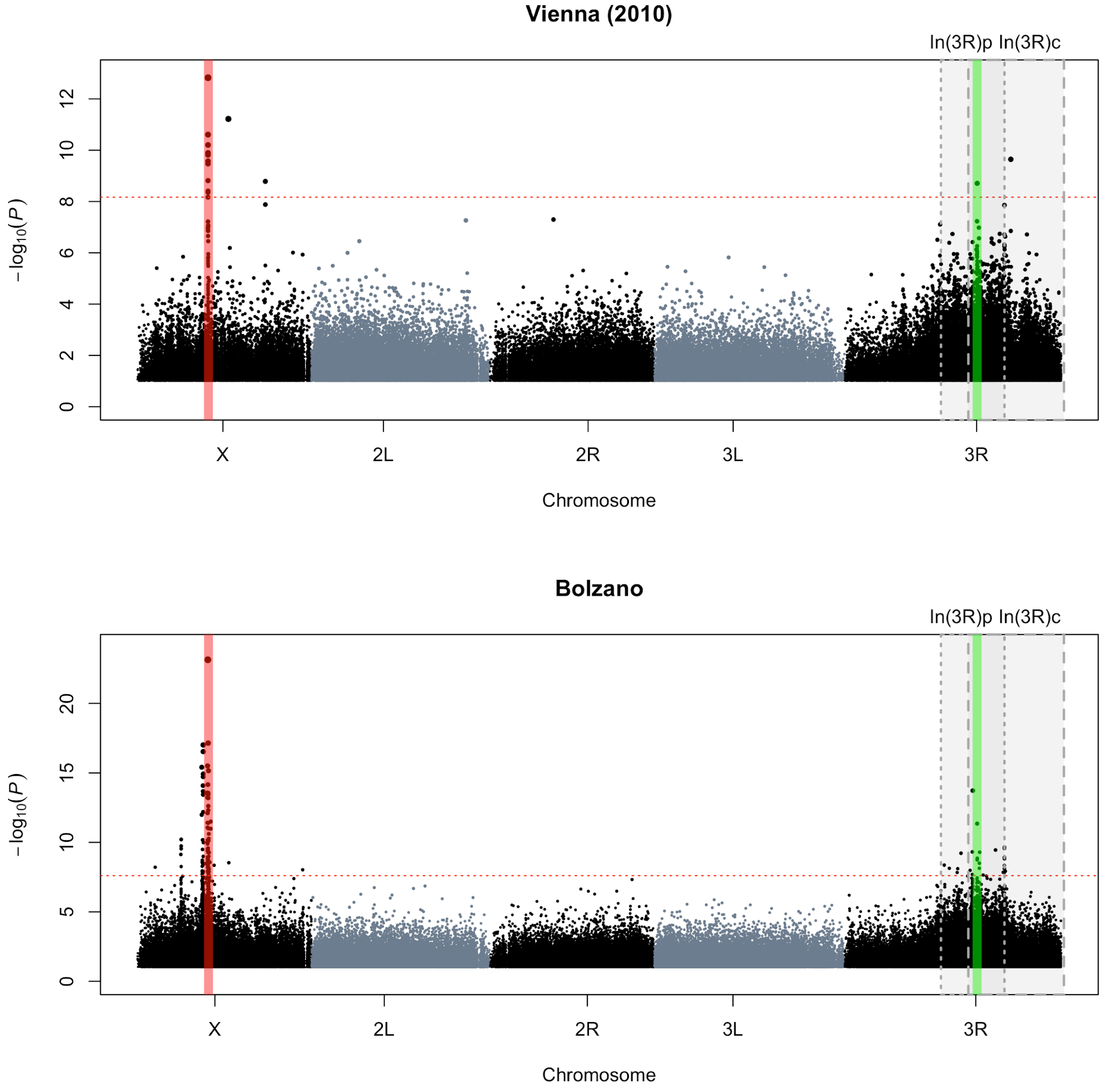

Figure S2: Manhattan plots of the of the association of SNPs with trident pigmentation in for the two Viennese replicates (upper plot) and the three Italian replicates (lower plot) as in Figure 2. The y-axis shows the negative decadic logarithm of the *p*-value of the CMH test, with the dotted red line indicating the empirical 5% FDR threshold. The region around *tan* is highlighted in red, the region around *ebony* in green. The grey rectangles indicate the location of the three most strongly associated cosmopolitan inversions. Counts for SNPs on autosomes were downsampled to 50% to make them directly comparable to the X chromosome.

MOLECULAR ECOLOGY

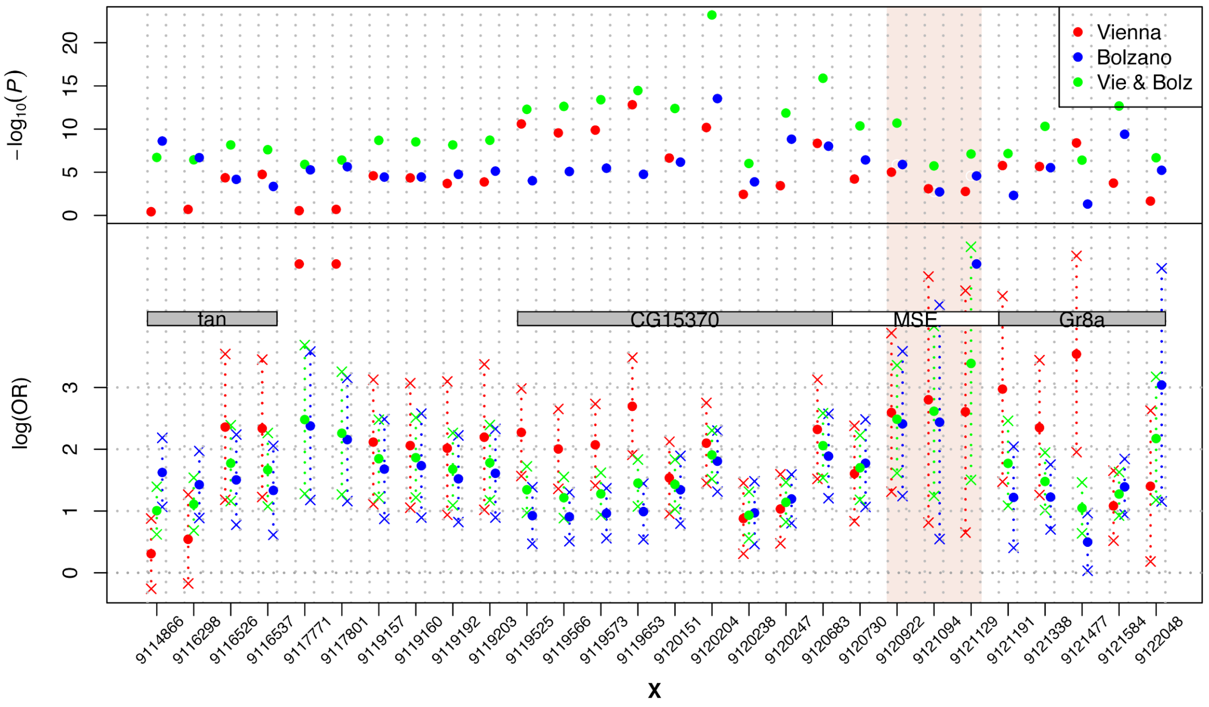

Figure S3: Display of the most highly associated variants around the *tan* locus. In the upper panels the upper panel the negative decadic logarithm of the p-values of the SNPs are shown, in the lower the natural logarithm of the estimated odds ratio of the dark-allele counts in the dark versus the light pools is shown (log(OR), dashed lines indicate 95% confidence intervals) as a proxy to the effect size. Grey rectangles indicate the location of genes, a white box that of the male specific enhancer (tMSE). The different colors indicate the populations used for the association tests (red: Vienna, blue: Bolzano, green: both populations combined). The pink region highlights the three variants in the *tMSE* region studied in the transgenic flies.

MOLECULAR ECOLOGY

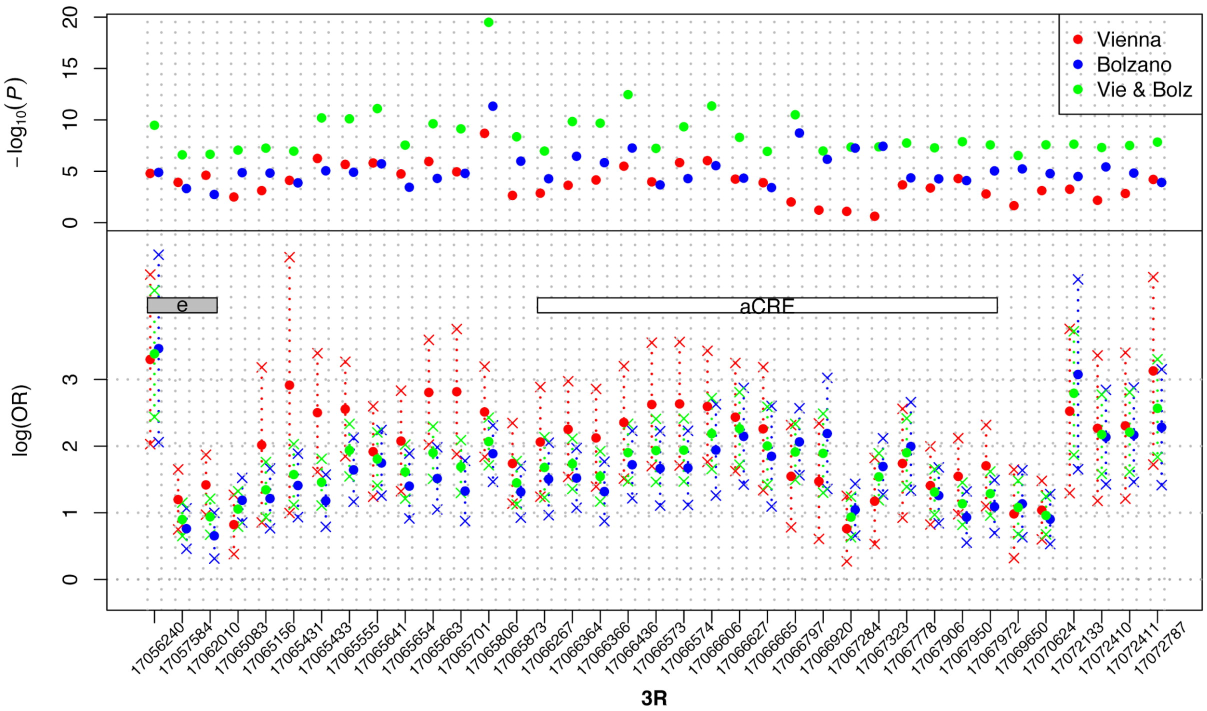

Figure S4: Display of the most highly associated variants around the *ebony* locus. In the upper panels the upper panel the negative decadic logarithm of the p-values of the SNPs are shown, in the lower the natural logarithm of the estimated odds ratio of the dark-allele counts in the dark versus the light pools is shown (log(OR), dashed lines indicate 95% confidence intervals) as a proxy to the effect size. Grey rectangles indicate the location of genes (e: *ebony*), a white box shows the abdominal cis-regulatory element (aCRE). The different colors indicate the populations used for the association tests (red: Vienna, blue: Bolzano, green: both populations combined).

MOLECULAR ECOLOGY

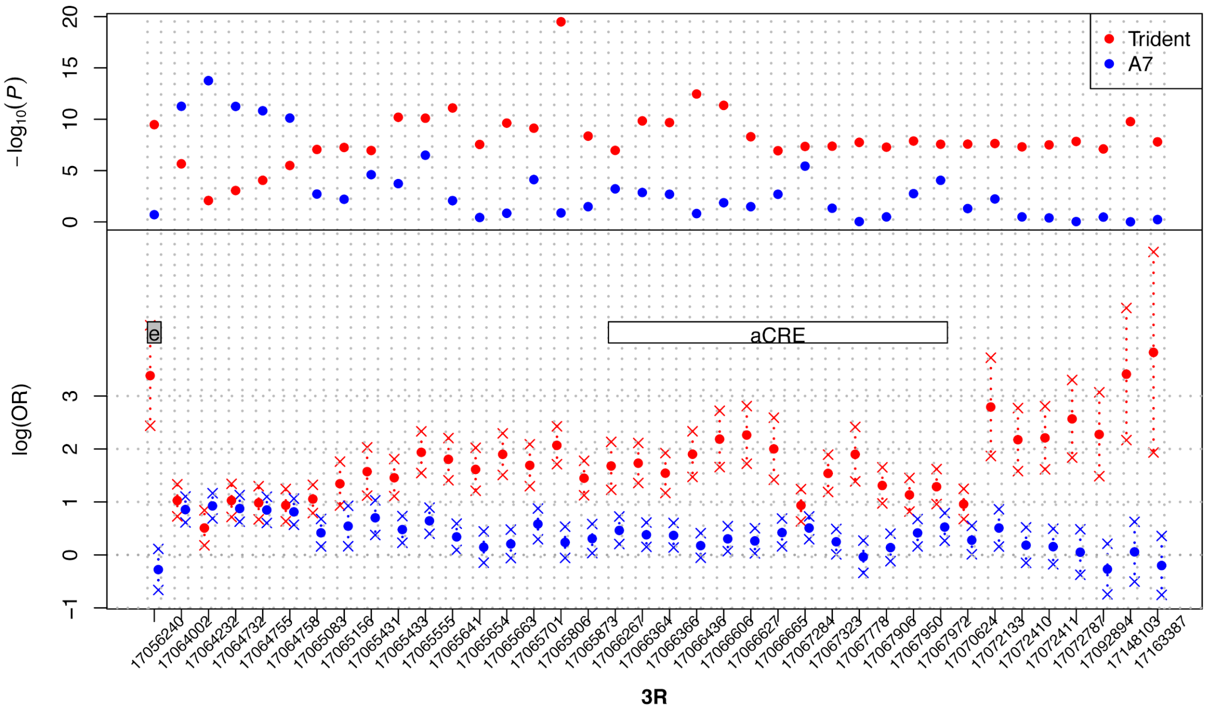

Figure S5: Comparison of the most highly associated SNPs in the trident Pool-GWAS (red) with the results of the female abdominal pigmentation GWAS (A7, blue (Endler et al., 2016)) around the *ebony (e)* locus. As in Figure 3, in the upper panel the negative decadic logarithm of the p-values of the SNPs are shown, in the lower the natural logarithm of the estimated odds ratio of the dark-allele counts in the dark versus the light pools is shown (log(OR)) as a proxy to the effect size. Grey rectangles indicate the location of genes, white of the abdominal cis-regulatory element (aCRE). Only SNPs with a *p*-value below $10^{-6.8}$ for trident or 10^{-10} for female abdominal pigmentation are displayed. A higher *p-value* than the 5% FDR threshold was used for the abdominal data to show the most highly associated variants.

MOLECULAR ECOLOGY

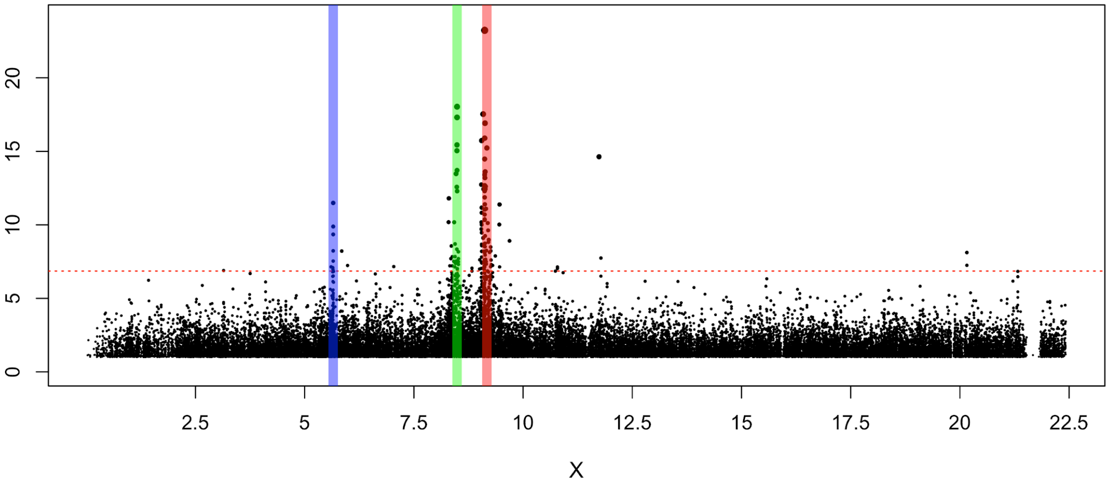

Figure S6: Manhattan plots of the of the association of SNPs with trident pigmentation as in Figure 2 showing only the X chromosome. The y-axis shows the negative decadic logarithm of the *p*-value of the CMH test, with the dotted red line indicating the empirical 5% FDR threshold. The region around *tan* is highlighted in red, the region around *Crag* in green and the region around *CG15771* and *CG15772* in blue.

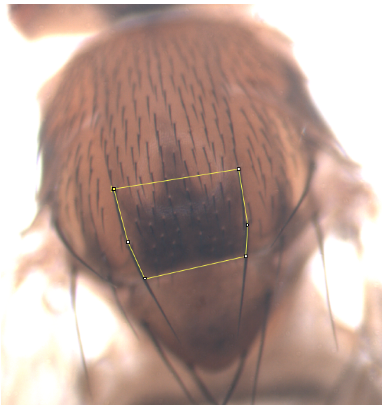

Figure S7: Example of the polygonic area (yellow) used for quantifying pigmentation intensity with ImageJ.

MOLECULAR ECOLOGY

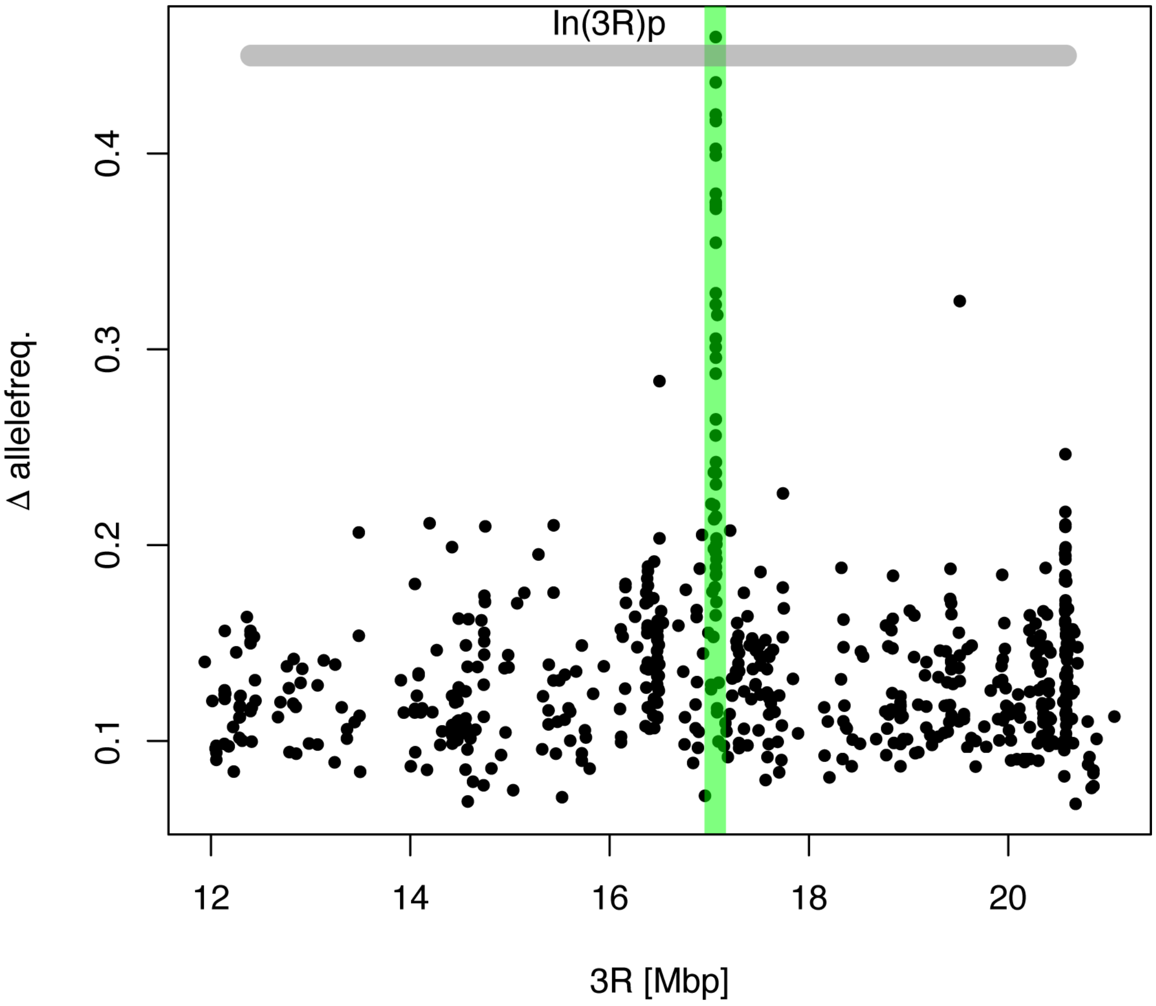

Figure S8: Allele frequency change of the light alleles between the dark and light pools averaged over all replicates. Only significantly associated variants in the region overlapping *In(3R)p* +/- 500000 bp were considered. The green line indicates the *ebony* locus, the grey bar the extent of the inversion *In(3R)p*.

MOLECULAR ECOLOGY

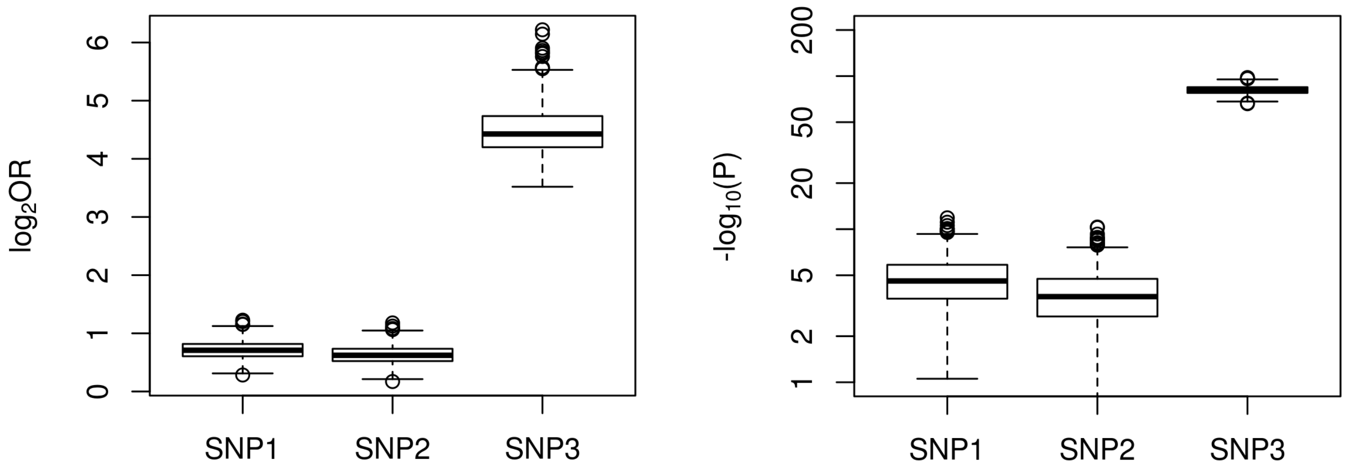

Figure S9: Per SNP $\log_2$OR (left) and $-\log_{10}$(P) (right) of 1000 simulated *in silico* Pool-GWAS experiments using the haplotype frequencies of SNPs 1, 2 & 3 identified in the European populations using the paired end read data (supplementary Table S4) and the pigmentation scores obtained from the transgenic assays with a heritability of h^2=0.2. For the CMH test five replicates of 1500 individuals with a light pool of 100 and a dark pool of 75 individuals were simulated. As SNP3 showed extremely low p-values in the simulations, the scale of the $-\log_{10}$(P) axis in the right panel is logarithmic.
